# Supplementary material for: A Global Screen for Assembly State Changes of the Mitotic Proteome by SEC-SWATH-MS
Source: Cell Syst. 2020 Feb 26;10(2):133–155.e6. doi: 10.1016/j.cels.2020.01.001 (PMC7042714; doi:10.1016/j.cels.2020.01.001)

Q9Y2R0 | COA3_HUMAN | COA3 CCDC56 MITRAC12 HSPC009
Monomer MW [kDa]: 11.731 Monomer expected elution fraction: 59

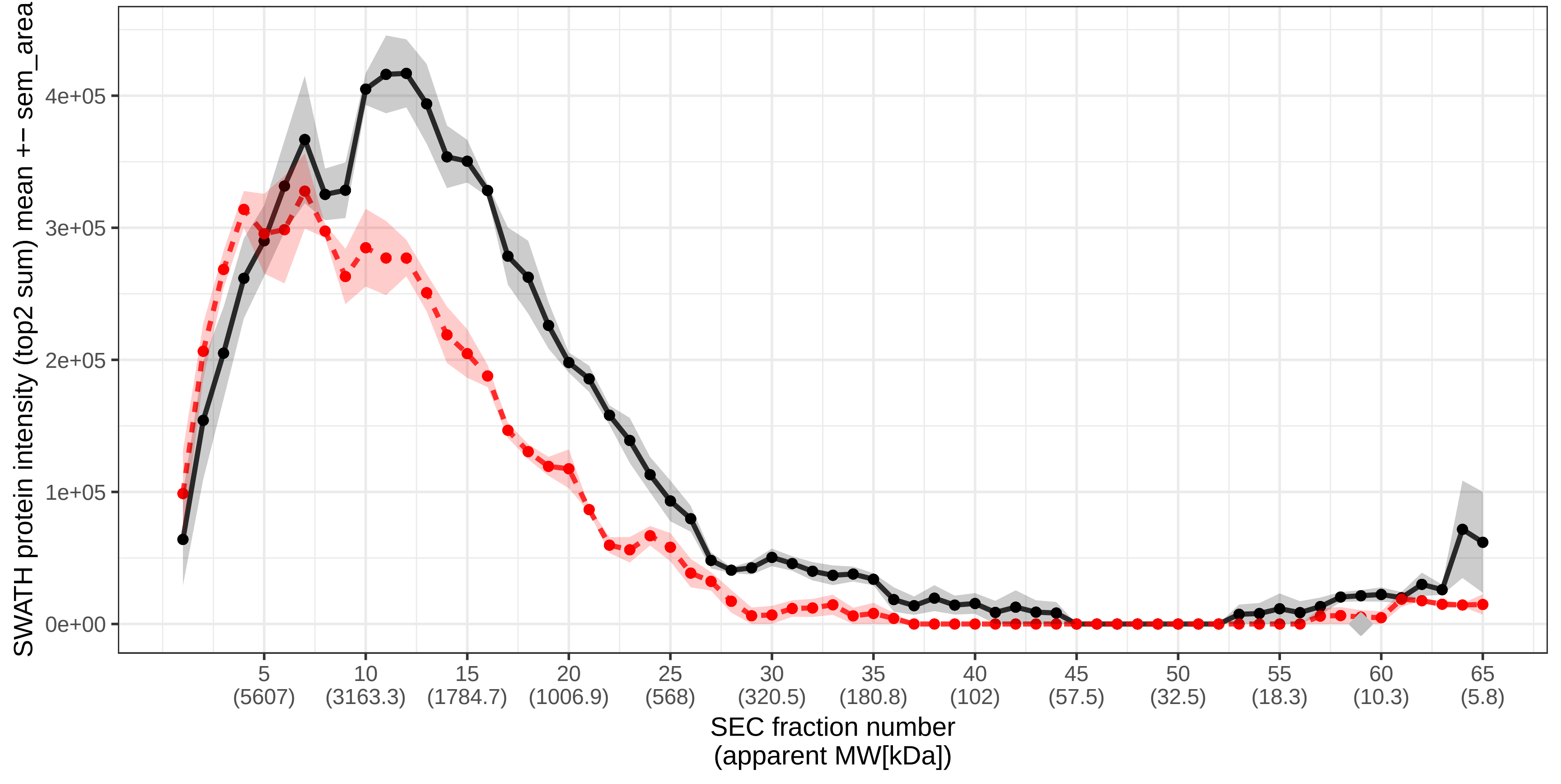

Supplement: Data S1. SEC-SWATH-MS Protein Chromatograms, Related to Figure 1 [file mmc6.zip › SECchrom_Q9Y2R0_COA3_HUMAN_COA3_CCDC56_MITRAC12_HSPC009.pdf]
